# Association between metformin use and mortality in patients with type 2 diabetes mellitus and localized resectable pancreatic cancer: a nationwide population-based study in korea

**DOI:** 10.18632/oncotarget.14525

**Published:** 2017-01-05

**Authors:** Won Il Jang, Mi-Sook Kim, Shin Hee Kang, Ae Jung Jo, Yun Jung Kim, Ha Jin Tchoe, Chan Mi Park, Hyo Jeong Kim, Jin A Choi, Hyung Jin Choi, Eun-Kyung Paik, Young Seok Seo, Hyung Jun Yoo, Jin-Kyu Kang, Chul Ju Han, Yeon Ju Kim, Sang Beom Kim, Min Jung Ko

**Affiliations:** ^1^ Department of Radiation Oncology, Korea Institute of Radiological and Medical Sciences, Seoul 01812, Republic of Korea; ^2^ Division for Healthcare Technology Assessment Research, National Evidence-based Healthcare Collaborating Agency, Seoul 04554, Republic of Korea; ^3^ Department of Anatomy, Seoul National University College of Medicine, Seoul 03080, Republic of Korea; ^4^ Department of Internal Medicine, Korea Institute of Radiological and Medical Sciences, Seoul 01812, Republic of Korea; ^5^ Department of Surgery, Korea Institute of Radiological and Medical Sciences, Seoul 01812, Republic of Korea

**Keywords:** pancreatic cancer, resection, diabetes, metformin, nationwide database

## Abstract

**Background:**

Preclinical studies support an antitumor effect of metformin. However, clinical studies have conflicting results and metformin's effect remains controversial. The aim of this study was to evaluate metformin's effect on clinical outcomes in diabetic patients with pancreatic cancer treated with curative resection.

**Results:**

A total of 764 patients underwent curative resection, met none of the exclusion criteria, and were prescribed oral hypoglycemic agents. The cancer-specific survival (5-year, 31.9% vs. 22.2%, *p* < 0.001) was significantly higher in the 530 metformin users than in the 234 diabetic metformin non-users. After multivariable adjustments, metformin users had significantly lower cancer-specific mortality as compared with metformin non-users (hazard ratio, 0.727; 95% confidence interval, 0.611–0.868). Cubic spline regression analysis demonstrated significantly decreased cancer-specific mortality with increasing dose of metformin (*p* = 0.0047).

**Materials and Methods:**

Data were provided from the Korea Central Cancer Registry and the National Health Insurance Service in the Republic of Korea. The study cohort consisted of 28,862 patients newly diagnosed with pancreatic cancer between 2005 and 2011. Metformin exposure was determined from prescription information from 6 months before the first diagnosis of pancreatic cancer to last follow-up. The main outcome was cancer-specific survival.

**Conclusions:**

This large study indicates that metformin might decrease cancer-specific mortality rates in localized resectable pancreatic cancer patients with pre-existing diabetes, independently of other factors, with a dose-response relationship.

## INTRODUCTION

Pancreatic cancer has become the seventh leading cause of cancer mortality in the world. In 2012, 337,872 people worldwide were diagnosed with pancreatic cancer, and 330,391 people died of this cancer [1]. The prognosis for patients with pancreatic cancer remains extremely dismal, with a 5-year relative survival rate of only 7% [2]. Less than 20% of patients present with localized disease eligible for curative surgery, and local recurrence (> 20%) and distant metastasis (> 70%) frequently occur after resection. The actuarial 5-year overall survival of patient who present with localized disease is only 20–25% due to the lack of effective adjuvant treatment strategies for this malignancy [3, 4].

The relationships between diabetes and pancreatic cancer are especially complicated and intertwined. About 80% of pancreatic cancer patients have either glucose intolerance or diabetes [5]. Pancreatic cancer is thought to causes diabetes, although the mechanism is not yet completely understood. On the other hand, diabetes appears to be a risk factor for the development of pancreatic cancer [6]. Diabetes may affect treatment outcomes of patients with pancreatic cancer, although the evidence is not consistent [7–9]. Furthermore anti-diabetic medications have been reported to affect pancreatic cancer risk. Sulfonylureas are associated with an increased risk of pancreatic cancer [10]. On the other hand, metformin usage has been found to be associated with a reduced risk of pancreatic cancer [11].

Metformin (1,1-dimethylbiguanide hydrochloride), one of the most widely prescribed drugs for type 2 diabetes mellitus, has been shown to be clinically associated with antitumor effects [12, 13]. Through a number of population, epidemiologic, and cohort studies, metformin has been suggested not only to prevent development of various tumors but also to delay cancer progression in certain tumor types [14–17]. In pancreatic cancer, substantial preclinical studies of metformin support metformin's ability to inhibit tumorigenesis and their authors have proposed potential mechanisms of the antitumor effect [18–21]. However, a few clinical studies have presented conflicting results and metformin`s effects in pancreatic cancer remain controversial [9, 21–24].

Hence, we hypothesized that the use of metformin might be associated with survival benefits for patients with resectable pancreatic cancer. We conducted a cohort study based on a nationwide population database to evaluate the effect of metformin on the clinical outcomes in patients with pre-existing diabetes and pancreatic cancer treated with curative resection.

## RESULTS

**Figure 1:**
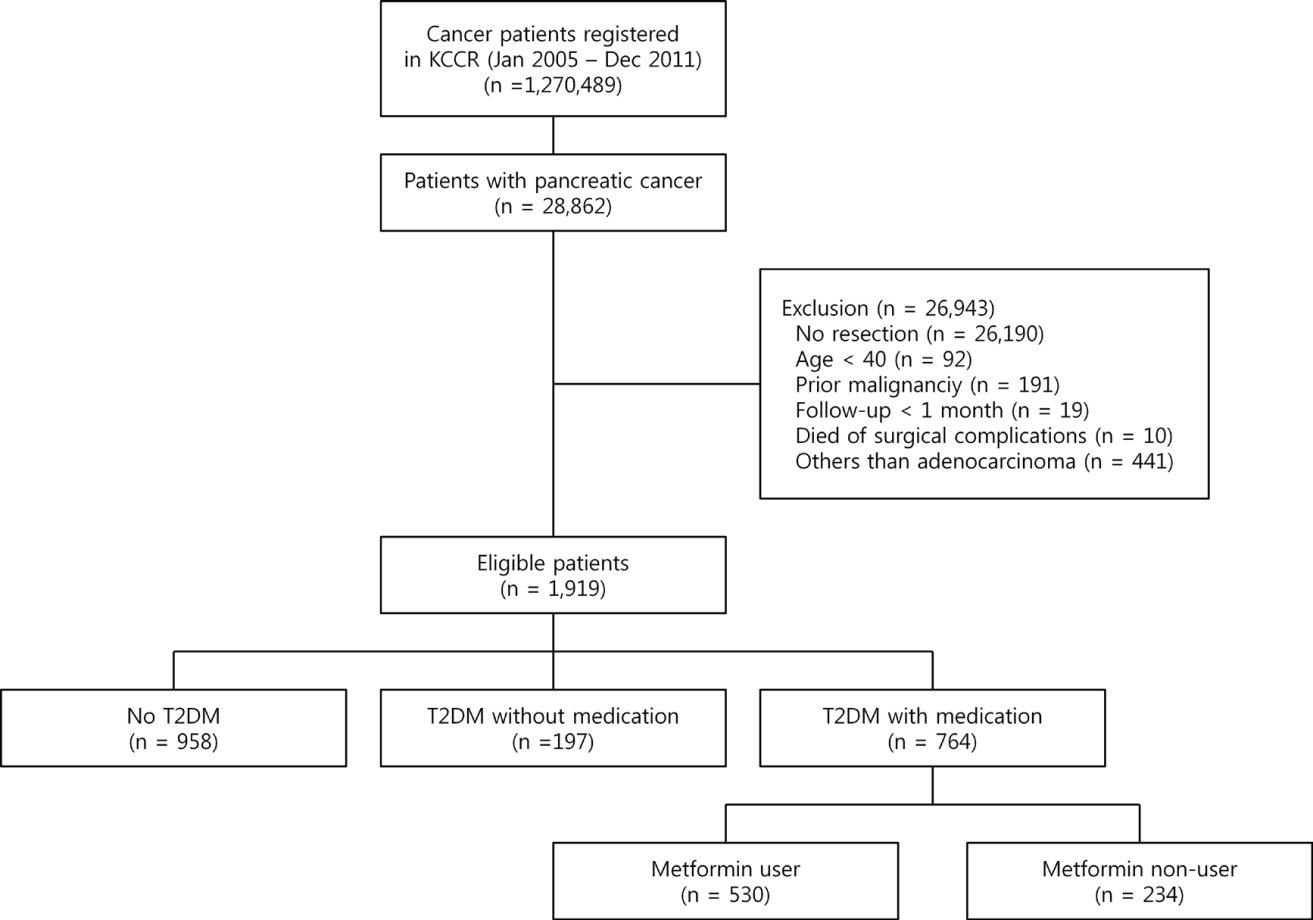
Flow diagram of the study KCCR, Korea Central Cancer Registry; DM, diabetes mellitus.

Among 28,862 pancreatic cancer patients who were diagnosed between January 1, 2005 and December 31, 2011, 1,919 patients met the eligibility criteria described in Figure 1. Of them, 764 patients were prescribed oral hypoglycemic agents (OHAs) for at least 90 days and classified as the diabetic group; 530 patients received metformin for at least 90 days (Figure 1). The median age was 65 years (interquartile range, 57–70) and 57.7% of the patients were men. The metformin non-user group had more current smokers, a higher prevalence of elevated aspartate transaminase (AST) and a lower prevalence of elevated alanine transaminase (ALT) when compared with the metformin user group. There were no significant difference between the metformin user group and non-user group in terms of age, sex, alcohol drinking behavior, regular exercise, body mass index (BMI), total cholesterol, fasting blood glucose, gamma-glutamyl transpeptidase (rGT), Charlson comorbidity index, treatment methods such as radiotherapy, chemotherapy, or types of surgery (Table 1).

**Table 1:** Baseline characteristics of the patients according to use of metformin as an initial pharmacotherapy

Variable		Metformin user (*n* = 530)	Metformin non-user (*n* = 234)	*p*-value
Demographic				
Age (years)	Median (Q1, Q3)	65 (57, 70)	65 (57, 70)	
	40–49	34 (6.4%)	17 (7.3%)	0.9033
	50–59	140 (26.4%)	60 (25.6%)	0.9072
	60–69	218 (41.1%)	97 (41.5%)	
	70–	138 (26.0%)	60 (25.6%)	
Sex	Male	306 (57.7%)	135 (57.7%)	0.9910
	Female	224 (42.3%)	99 (42.3%)	
*Health risk behavior				
Smoking status	Never smoker	225 (68.0%)	81 (60.0%)	
	Former smoker	51 (15.4%)	16 (11.9%)	0.0121
	Current smoker	55 (16.6%)	38 (28.1%)	
Alcoholic drinking	Yes	14 (4.2%)	9 (6.7%)	0.2706
	No	317 (95.8%)	126 (93.3%)	
Regular exercise	Yes	52 (15.7%)	23 (17.0%)	0.7236
	No	279 (84.3%)	112 (83.0%)	
*Laboratory				
Body mass index (kg/m2)	Median (Q1, Q3)	24.0 (21.9, 25.6)	24.0 (21.8, 25.8)	0.8515
	≥ 25	116 (35.0%)	48 (35.6%)	0.6698
	< 25	215 (65.0%)	87 (64.4%)	
Total cholesterol (mg/dl)	Median (Q1, Q3)	188 (158, 216)	186 (161, 217)	0.6448
	≥ 240	43 (13.0%)	11 (8.1%)	0.0898
	< 240	288 (87.0%)	124 (91.9%)	
Fasting blood glucose (mg/dl)	Median (Q1, Q3)	119 (101, 150)	108 (96, 121)	0.0618
	≥ 126	140 (42.3%)	30 (22.2%)	0.8127
	< 126	191 (57.7%)	115 (77.8%)	
AST (U/L)	Median (Q1, Q3)	25 (20, 35)	25 (20, 32)	0.6586
	≥ 51	36 (10.9%)	17 (12.6%)	0.0091
	< 51	295 (89.1%)	118 (87.4%)	
ALT (U/L)	Median (Q1, Q3)	24 (17, 37)	22 (17, 32)	0.2034
	≥ 46	55 (16.6%)	13 (9.6%)	0.0310
	< 46	276 (83.4%)	122 (90.4%)	
rGT (U/L)	Median (Q1, Q3)	29 (19, 51)	30 (20, 51)	0.4752
	≥ 78 (male), 46 (female)	61 (18.4%)	29 (21.5%)	0.7269
	< 78 (male), 46 (female)	270 (81.6%)	106 (78.5%)	
Comorbidity				
Charlson comorbidity index	Median (Q1, Q3)	6 (5, 8)	6 (5, 8)	
	0–6	278 (52.5%)	119 (50.9%)	0.5528
	7–9	172 (32.5%)	82 (35.0%)	0.7729
	≥ 10	80 (15.1%)	33 (14.1%)	
Methods of treatment				
Radiotherapy	Yes	168 (31.7%)	80 (34.2%)	0.4981
	No	362 (68.3%)	154 (65.8%)	
Chemotherapy	Yes	348 (65.7%)	165 (70.5%)	0.1881
	No	182 (34.3%)	69 (29.5%)	
Type of surgery	Whipple/PPPD	333 (62.8%)	159 (67.9%)	0.1732
	Distal pancreatectomy	197 (37.2%)	75 (32.1%)	
Antidiabetic medications except metformin				
Kinds of medications	Sulfonylurea	457 (86.2%)	192 (82.1%)	0.1369
	Insulin	326 (61.5%)	131 (56.0%)	0.1509
	Thiazolidinedione	115 (21.7%)	44 (18.8%)	0.3636
	DPP-4 inhibitor	90 (17.0%)	9 (3.8%)	< 0.0001
	Others	332 (62.6%)	150 (64.1%)	0.6997

Q1, the first quartile; Q3, the third quartile; AST, aspartate transaminase; ALT, alanine transaminase; Rgt, gamma-glutamyl transpeptidase; PPPD, pylorus preserving pancreaticoduodenectomy; DPP-4, dipeptidyl peptidase-4.

* Health risk behavior and laboratory data were available for 331 and 135 patients in the metformin user and the non-user group, respectively. Missing data were not imputed.

**Figure 2:**
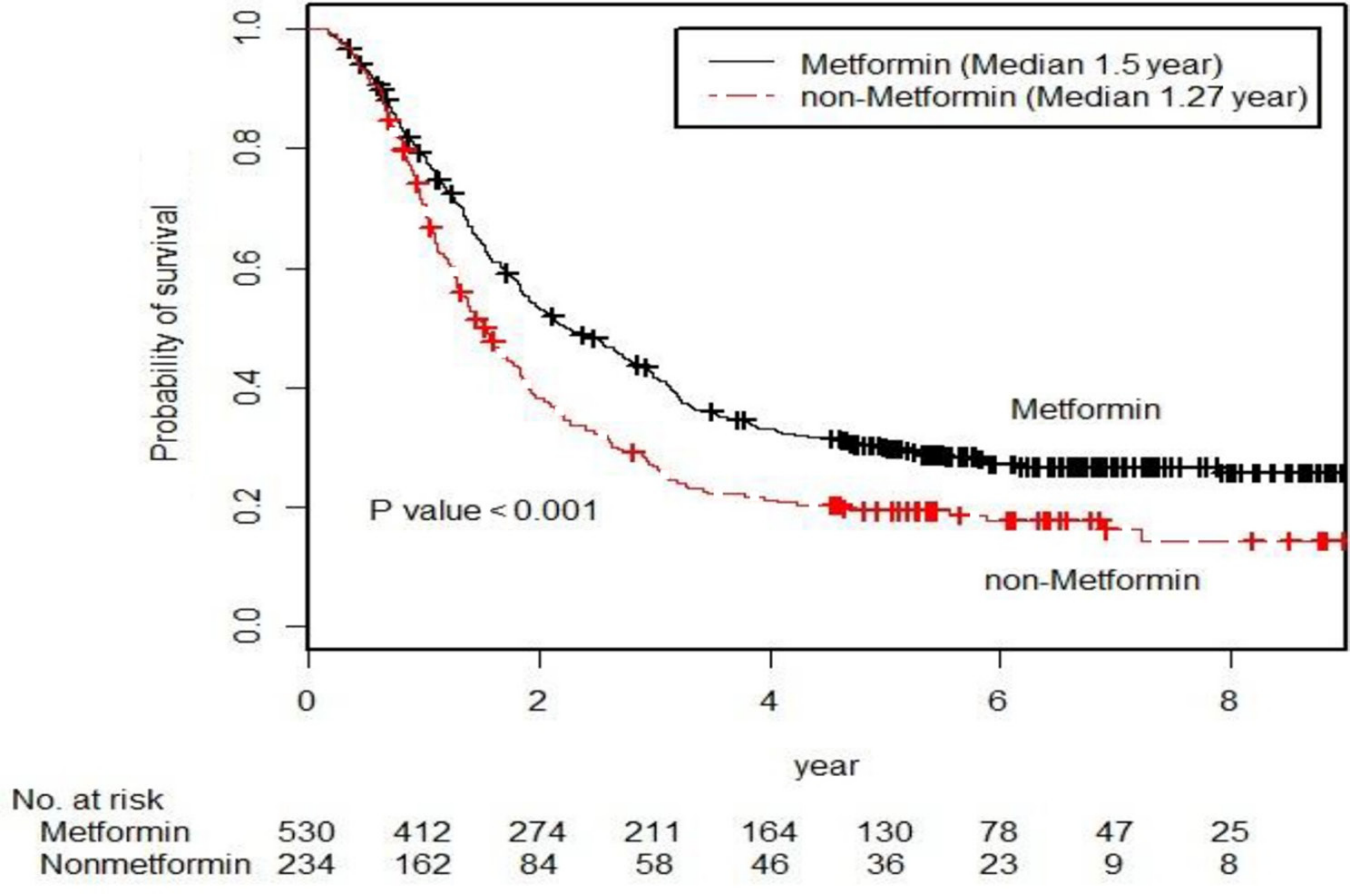
Kaplan-Meier survival curve for the cancer-specific survival of the metformin user group and the metformin non-user group (*p*-values by the log-rank test)

**Figure 3:**
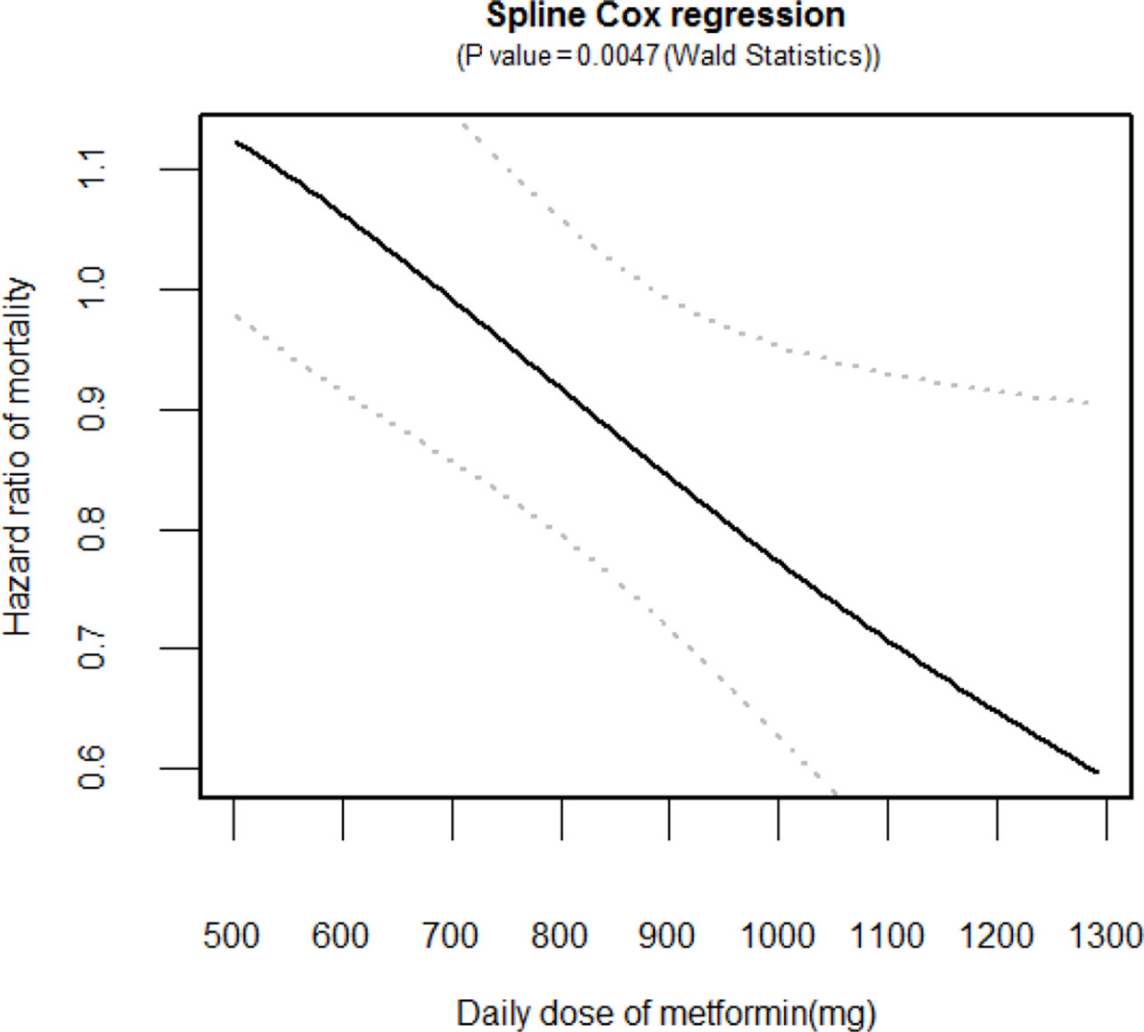
Dose-response relationship between an exposure dose of metformin and cancer-specific mortality Hazard ratio (solid line) and 95% confidence interval (dashed lines) are from the cubic spline regression model.

The cancer-specific survival (5-year, 31.9% vs. 22.2%, *p* < 0.001 by the log-rank test) was significantly higher in the metformin user group than in the metformin non-user group among the diabetic groups during the follow-up period (Figure 2). In unadjusted analyses, compared to the metformin non-user group, the metformin user group showed a significantly lower risk of cancer-specific mortality (hazard ratio [HR], 0.702; 95% confidence interval [CI], 0.588–0.837). After multivariable adjustments for clinical covariates, the metformin user group still had a significantly lower risk of events as compared with the metformin non-user group (HR, 0.727; 95% CI, 0.611–0.868) (Table 2). In the metformin user group, the adjusted risk for cancer-specific mortality was significantly lower for patients with an medication possession ratio (MPR) ≥ 80% compared to those with an MPR < 80% (HR, 0.595; 95% CI, 0.468–0.757) (Table 2). In the dose-response relationship analysis, we modeled the association between an exposure dose of metformin and cancer-specific mortality using a cubic spline regression model. The negative linear dose-response trend demonstrated a statistically significant decreased cancer-specific mortality with increasing exposure dose of metformin. The cancer-specific mortality was almost 43% lower (HR, 0.668; 95% CI, 0.529–0.845) for those who received more than 1000 mg metformin daily and compared to the metformin non-user group (Figure 3).

**Table 2:** Pancreatic cancer-specific mortality and hazard model according to use of metformin and medication possession ratio (MPR)

Group	No. of patients	Pancreatic cancer-specific mortality		
		No. of event (%)	Unadjusted HR (95% CI)	*Adjusted HR (95% CI)
Metformin user	530	373 (70.4%)	0.702 (0.588–0.837)	0.727 (0.609–0.868)
Metformin non-user	234	186 (79.5%)	1	1
MPR ≥ 80 in metformin user	152	88 (57.9%)	0.592 (0.466–0.753)	0.595 (0.468–0.757)
MPR < 80 in metformin user	378	285 (75.4%)	1	1

HR, hazard ratio; CI, confidence interval.

*Adjusted with age, sex, and Charlson comorbidity index. Hazard ratios were calculated with Cox proportional hazards model for patients with metformin user with reference to patients with metformin non-users. All statistical tests were two-sided.

In sensitivity analyses, the risks for cancer-specific mortality were consistently lower in the metformin user group when we confined this analysis to those who initiated their prescription during the 6 months before diagnosis, during the 6 months before and after diagnosis, or during the 6 months before and the 12 months after diagnosis. In addition, these lower risks of the metformin user group for cancer-specific mortality were also found in the second sensitivity analysis performed among those whose complete health examination data were available. Similar lower risks of the metformin user group for cancer-specific mortality were shown in the third sensitivity analyses performed among patients treated with chemotherapy, those treated with radiotherapy, or those treated with pancreatic head resection such as Whipple`s procedure or p ylorus-preserving pancreaticoduodenectomy (Table 3).

**Table 3:** Sensitivity analyses of the association between use of metformin and pancreatic cancer-specific mortality

Variable	No. of patients	Pancreatic cancer-specific mortality		
		*Adjusted HR	95% CI	*p*-value
Initiation of metformin exposure				
During 6 months before diagnosis	408	0.771	0.621–0.956	0.0178
During 6 months before and 6 months after	574	0.744	0.616–0.899	0.0022
During 6 months before and 12 months after	651	0.740	0.616–0.889	0.0013
Complete health examination data	466	0.714	0.553–0.923	0.0101
Chemotherapy	513	0.669	0.544–0.821	0.0001
Radiotherapy	248	0.736	0.547–0.990	0.0426
Whipple`s procedure or PPPD	492	0.796	0.644–0.984	0.0350
Distal pancreatectomy	272	0.576	0.419–0.792	0.00007

HR, hazard ratio; CI, confidence interval; PPPD, pylorus preserving pancreaticoduodenectomy.

* Adjusted with age, sex, and Charlson comorbidity index, hazard ratios were calculated with Cox proportional hazards model for patients with metformin user with reference to patients with metformin non-users. All statistical tests were two-sided.

## DISCUSSION

In the present study, we found that those receiving metformin have lower cancer-specific mortality rates than those not receiving metformin in localized resectable pancreatic cancer patients with pre-existing diabetes. In addition, metformin usage was independently predictive of cancer-specific mortality after multivariable adjustment for clinical covariates. This finding is not caused by a difference in treatment methods, because these were balanced between the two pancreatic cancer groups with pre-existing diabetes and our findings remained the same after restricting treatment methods from the analyses. This is the first study showing beneficial effects of metformin in patients with localized resectable pancreatic cancer.

Although an antitumor effect of metformin has been shown in preclinical studies and population analyses, several cohort studies have not shown a consistent survival benefit from metformin in pancreatic cancer patients with pre-existing diabetes [9, 21–24]. Sadeghi et al. showed that metformin usage is significantly associated with longer survival in patients with non-metastatic disease only [22], but that benefit was not significant in those with metastatic disease. In a subclass analysis of patients with non-metastatic disease, resectable disease was not associated with a survival benefit, which may be explained by the small sample size, including only 22% of the study population. Choi et al. showed that metformin usage is associated with a longer overall survival for advanced pancreatic cancer patients receiving palliative chemotherapy [9]. However, these studies, based on retrospective analysis of single institution data, were associated with limitations of small sample sizes and uncontrolled selection bias. Recently, a randomized phase 2 trial was performed to find the synergism between metformin and chemotherapeutic agents in metastatic or unresectable locally advanced pancreatic cancer. They reported that metfromin was not beneficial as an add-on to chemotherapy [21]. In fact, the lack of efficacy of metformin in these patients with advanced disease may be explained by a small study sizes and a modest anti-tumor effect of metformin, compounding by heterogeneous prognostic factors, large tumor burden, and a potential difference in tumor biology in metastatic or unresectable locally advanced disease. Unlike these studies, only patients receiving curative resection for localized pancreatic cancer were included in our study to minimize the mixed effect from differing extents of disease. Resectable pancreatic cancer has been considered to be relatively homogeneous population with the similar prognosis [25–27]. Although we could not get more detailed staging information than the Surveillance, Epidemiology, and End Results (SEER) summary stage in the KCCR database, it is quite reasonable to include only resectable pancreatic cancer. It may only have been possible to show these results because we obtained sufficient number of cases of pancreatic cancer from a linked nationwide database. Therefore, our results suggest any prospective randomized trial should be performed with large number of patients and be stratified by treatment options to obtain a homogenous cohort.

Findings from our study should be interpreted in the context of the following limitations. Firstly, confounding by indication is an intractable threat to validity in observational studies, although we used adjusted models to account for confounding factors. Potential confounding may exist due to the lack of some data, such as hemoglobin A1c level, carbohydrate antigen 19–9, tumor size, lymph node status, or surgical margin status [28, 29]. Secondly, immortal person time is a major concern in these types of observational study [30–32]. We conducted sensitivity analyses to minimize immortal person time bias and the difference in metformin initiation time. In sensitivity analyses, the risks for mortality were consistently lower in the metformin user group, even when we confined this analysis to those who initiated metformin prescription during the 6 months before diagnosis, during the 6 months before and after diagnosis, or during the 6 months before and the 12 months after diagnosis. Thirdly, beneficial effects of metformin, which is the most widely used first-line type 2 diabetes medication, may be due to use of metformin among healthier patients with early diabetes. On the contrary, patients using insulin may have poor glucose control from oral medications or a poor performance status which preclude the use of oral medications [28]. In another sensitivity analysis to minimize healthy user bias, we demonstrated that metformin usage was associated with better survival outcome, even when compared with non-diabetic control patients, despite a preponderance of insulin users in the metformin user group. Fourthly, this study included only Korean population, which is quite homogeneous. There may be differences, such as BMI or any other ethnic-specific factors, among Korean patients with pancreatic cancer and others ethnic groups. The SEER research data demonstrated differences among different ethnic groups with pancreatic cancer in the United States with respect to both incidence and survival [33]. For universal applicability, further studies on other ethnic groups are needed. In conclusion, our study indicates that metformin might decrease cancer-specific mortality rates of localized resectable pancreatic cancer patients with pre-existing diabetes, independently of other factors, by means of a dose-response relationship. Considering the high prevalence of diabetes in patients with pancreatic cancer and the lack of effective treatment strategies for this malignancy, well-designed prospective studies are warranted to confirm the survival benefit of metformin in patients with diabetes and resectable pancreatic cancer. If proven beneficial, metformin may be an ideal adjuvant treatment option because it is inexpensive, safe, and well tolerated. We hope that our study can serve as a proof-of-concept for prospective studies of metformin to prevent or defer recurrence and to prolong survival in patients with resectable pancreatic cancer.

## MATERIALS AND METHODS

### Data sources

The data were provided by the KCCR and they were linked to national claims data from the NHIS through the use of unique personal identification numbers with the consent of KCCR. The data from the KCCR covers nationwide cancer cases in Korea, and it includes patients’ date and site of primary cancer diagnosis [34]. The NHIS covers 98% of the Korean population for the whole lifespan, and the NHIS has comprehensive data sets for diagnoses, treatments, procedures, surgical history, prescription records, and periodic general health examinations data of all insured patients. In addition, we obtained the cause and date of death from the NPR of the Korea NSO, with the use of the unique personal identification numbers. IRB waived the need for written informed consent from participants, because this study was based on routinely collected administrative data and why This study was approved by the Institutional Review Board of the National Evidence-based Healthcare Collaborating Agency (NECA IRB 14-004-1).

### Study subjects

The study population included 28,862 adults who had a primary diagnosis of pancreatic cancer (the International Classification of Diseases, 10th revision (ICD-10), C25) recorded by the KCCR between January 1, 2005 and December 31, 2011. Only individuals, who had undergone curative resection for localized pancreatic cancer, were eligible. The Whipple`s procedure, p ylorus-preserving pancreaticoduodenectomy, or distal pancreatectomy was defined as curative resection. Those, whose surgery was aborted due to findings of unresectability at the time of surgery, were excluded. The individuals with the age of 40 or less were excluded due to the possibility of genetic or family syndrome. The individuals were excluded if they had a history of invasive cancers other than pancreatic cancer or a follow-up duration of less than 1 month, died from complications following surgery, or were not diagnosed with pancreatic adenocarcinoma. The death from complications following surgery was defined as the death without evidence of disease progression within 1 month of the surgery date.

### Exposure and follow-up

The drug exposures of interest were metformin and non-metformin (sulfonylurea, insulin, thiazolidinedione, dipeptidyl peptidase 4 inhibitors, or other antidiabetic drugs). Drug exposure was defined as OHAs or insulin medication administration in the same class for at least 90 days in the period from 6 months before first diagnosis of pancreatic cancer to last follow-up. Participants were classified as patients with pre-existing diabetes and pancreatic cancer if they had a diagnosis of non-insulin-dependent diabetes (ICD-10, E11) and received the drug exposure on the basis of the above-mentioned definition. The MPR was defined as the total days the target medication was prescribed divided by the total days between prescriptions. An MPR of more than 80% was considered acceptable adherence. Cohort entry date for each patient was the date of first diagnosis of pancreatic cancer, and the exit date (censoring date) was the earliest of: a) date of death; b) date of recording of clinical event; c) 5 years after cohort entry or d) the end of the study period (December 31, 2013).

### Statistical analysis

In the main analysis, we compared the baseline characteristics and medications for OHA for patients with and without use of metformin. We further compared the event rates for cancer-specific mortality during the follow-up period between patients with and without metformin by cumulative probability curves derived from Kaplan-Meier estimates; the same analysis was applied for MPR groups among patients with metformin. After unadjusted analyses were initially performed, we conducted adjusted analyses, including the following potential confounders: age, sex, and Charlson comorbidity index [35]. We performed further analyses to compare metformin users with non-diabetic control patients to minimize healthy user bias. We performed cubic spline regression analysis to characterize a dose-response relationship between an exposure dose of metformin and cancer-specific mortality [36]. All analyses were performed with SAS software, version 9.3 (SAS Institute, Cary, NC). All reported p-values were 2-sided and a 5% or lower p-value was considered to be statistically significant.

### Sensitivity analyses

We conducted sensitivity analyses by the following restrictions. In the first sensitivity analysis, we restricted analysis among those whose exposure of metformin initiated from 180 days before pancreatic cancer diagnosis through the date of pancreatic cancer diagnosis to control immortal time bias [37]. In addition, information from the periodic general health examination was available for 61% of patients. These data included smoking status, alcohol drinking behavior, regular exercise, BMI, total cholesterol, fasting blood glucose, AST, ALT, and rGT. We performed the second sensitivity analyses among those with these data available. In the third sensitivity analyses, we restricted analysis among patients who received chemotherapy, radiotherapy, or pancreatic head resection such as Whipple`s procedure or p ylorus-preserving pancreaticoduodenectomy to obtain a homogeneous cohort. All of these patients received an adjuvant treatment and thus may regarded as representing a cohort that had more advanced diseases and better performance status.

## Abbreviations

BMI: body mass index
CI: confidence interval
ALT: alanine transaminase
AST: aspartate transaminase
rGT: gamma-glutamyl transpeptidase
HR: hazard ratio
ICD: International Classification of Diseases
KCCR: Korea Central Cancer Registry
MPR: medication possession ratio
NHIS: National Health Insurance Service
NPR: National Population Registry
NSO: National Statistical Office
OHA: oral hypoglycemic agent
SEER: Surveillance, Epidemiology, and End Results
